# Variation in *Mycobacterium tuberculosis* population structure in Iran: a systemic review and meta-analysis

**DOI:** 10.1186/s12879-020-05639-7

**Published:** 2021-01-04

**Authors:** Shima Hadifar, Abolfazl Fateh, Vahid Pourbarkhordar, Seyed Davar Siadat, Shayan Mostafaei, Farzam Vaziri

**Affiliations:** 1 Department of Mycobacteriology and Pulmonary Research, Pasteur Institute of Iran, Tehran, Iran; 2 Microbiology Research Centre (MRC), Pasteur Institute of Iran, Tehran, Iran; 3 Department of Pharmacology and Toxicology, School of Pharmacy and Pharmaceutical Science, Isfahan University of Medical Science, Isfahan, Iran; 4 Department of Biostatistics, School of Health, Kermanshah University of Medical Sciences, Kermanshah, Iran; 5 Epidemiology and Biostatistics Unit, Rheumatology Research Centre, Tehran University of Medical Sciences, Tehran, Iran

**Keywords:** *Mycobacterium tuberculosis*, Genotype, Diversity, Meta-analysis, Iran

## Abstract

**Background:**

Acquiring comprehensive insight into the dynamics of *Mycobacterium tuberculosis* (*Mtb*) population structure is an essential step to adopt effective tuberculosis (TB) control strategies and improve therapeutic methods and vaccines. Accordingly, we performed this systematic review and meta-analysis to determine the overall prevalence of *Mtb* genotypes/ sublineages in Iran.

**Methods:**

We carried out a comprehensive literature search using the international databases of MEDLINE and Scopus as well as Iranian databases. Articles published until April 2020 were selected based on the PRISMA flow diagram. The overall prevalence of the *Mtb* genotypes/sublineage in Iran was determined using the random effects or fixed effect model. The metafor R package and MedCalc software were employed for performing this meta-analysis.

**Results:**

We identified 34 studies for inclusion in this study, containing 8329 clinical samples. Based on the pooled prevalence of the *Mtb* genotypes, NEW1 (21.94, 95% CI: 16.41–28.05%), CAS (19.21, 95% CI: 14.95–23.86%), EAI (12.95, 95% CI: 7.58–19.47%), and T (12.16, 95% CI: 9.18–15.50%) were characterized as the dominant circulating genotypes in Iran. West African (L 5/6), Cameroon, TUR and H37Rv were identified as genotypes with the lowest prevalence in Iran (< 2%). The highest pooled prevalence rates of multidrug-resistant strains were related to Beijing (2.52, 95% CI) and CAS (1.21, 95% CI).

**Conclusions:**

This systematic review showed that *Mtb* populations are genetically diverse in Iran, and further studies are needed to gain a better insight into the national diversity of *Mtb* populations and their drug resistance pattern.

## Background

Tuberculosis (TB) remains the most lethal infectious disease with an estimated rate of 1.4 million deaths in 2018 [1]. Human-adapted *Mycobacterium tuberculosis* complex (MTBC), as a causative agent of TB infection, belong to eight phylogenetic branches with a phylogeographical population structure [2, 3]. These lineages include Indo-Oceanic lineage (Lineage 1), East Asian (Lineage 2), Central Asian (Lineage 3), Euro-American (Lineage 4), Ethiopian (Lineage 7), known as *Mycobacterium tuberculosis* sensu stricto, West African 1 (Lineage 5) and West African 2 (Lineage 6), referred to as *Mycobacterium africanum* and Lineage 8 (L8) which geographically restricted to the African Great Lakes region [2–4].

Different studies have shown that genomic differences among MTBC lineages or sublineages can affect the clinical and epidemiological characteristics of TB infection [5–8]. In recent decades, some *Mycobacterium tuberculosis* (*Mtb*) lineages/sublineages have attracted wide attention due to certain features such as transmission potential, pathogenic properties and association with drug resistance [9, 10]. Lineages 2 and 4 are widely distributed and seem to have a higher pathogenic power compared to geographically restricted lineages [2, 11, 12]. In West and South Asia, a sharp increase has been documented in the circulation of certain sublinages such as NEW-1 (Lineage 4) and CAS (Lineage 3) strains that are prone to emerging as resistant clones [13–15]. This growing increase seems be more important in Iran with the national average TB rate of 14 cases per 100,000 population, due to the influx of Afghan refugees and population growth [1]. Accordingly, acquiring comprehensive insight into the dynamics of *Mtb* population structure is an essential step to adopt effective TB control strategies and improve therapeutic methods and vaccines.

Therefore, the current systematic review and meta-analysis was conducted to determine (1) the overall prevalence of *Mtb* genotypes/sublineages and (2) to determine the dominant multidrug-resistant (MDR) *Mtb* genotypes in TB patients in Iran.

## Methods

### Study protocol

The meta-analysis was based on the PRISMA (Preferred Reporting Items for Systematic Reviews and Meta-Analyses) guidelines for systematic reviews and meta-analyses [16]. The study protocol was registered in the PROSPERO database (CRD42020186561).

### Search strategy and selection criteria

For evaluating the diversity of *Mtb* isolates in Iran, a comprehensive literature search was conducted using the international electronic databases of MEDLINE and Scopus as well as Iranian databases. English-language studies published until April 2020 were retrieved using the following keywords: “*Mycobacterium tuberculosis*”, “tuberculosis”, “molecular typing”, “genetic diversity”, “genotyping” and “Iran” combined with the Boolean operators “OR”, “AND” and “NOT” in the Title/Abstract/Keywords field. Additional keywords such as “lineage” combined with “*Mycobacterium tuberculosis*” were used to avoid missing any articles. Similar strategies using Persian keywords were used to find relevant Persian original articles in Iranian databases, such as Scientific Information Database (www.sid.ir), Irandoc (www.irandoc.ac.ir), Magiran (www.magiran.com), and Iranmedex (www.iranmedex.com).

The titles and abstracts of all the identified articles were reviewed for eligibility, then screening for relevant articles were performed by reviewing the full texts.

The inclusion criteria were: 1) studies reporting the prevalence of *Mtb* genotypes among TB patients, 2) studies presenting data from Iran irrespective of the publication year, and 3) studies used Spoligotyping, mycobacterial interspersed repetitive unit-variable number tandem repeat (MIRU-VNTR) typing and Whole-Genome Sequencing (WGS) methods for genotyping. The exclusion criteria, on the other hand, included:1) studies only presenting prevalence data on *Mtb* genotypes among drug-resistant *Mtb* isolates, 2) studies providing incomplete data, 3) studies published as meta-analyses and systematic reviews, 4) studies not in English or Persian, 5) studies limited to a single genotype, 6) studies that lacked genotyping data, and 7) studies that were not related to human TB molecular epidemiology. Data screening was performed by two reviewers independently.

### Data extraction and quality assessment

Data from the studies meeting our inclusion criteria were extracted. We required the following data: first author’s name, year of publication, study area, molecular techniques, genotype, number of genotypes, total sample size, MDR genotype, sample type and nationality.

According to the items defined in the Strengthening the Reporting of Observational Studies in Epidemiology (STROBE) checklist, we evaluated the methodological quality of the included studies using the pre-defined criteria presented in Table 1. This checklist consists of various methodological aspects, and a maximum quality evaluation score of 32 was considered and articles with scores below 18 were excluded from this study [51]. Data extraction and quality assessment were also carried out by two reviewers independently.

**Table 1:** Characteristics of 34 included studies in this meta-analysis

First author	Location	Typing method	Year of publication	Genotypes	Total of genotypes	MDR genotypes	Sample size	STROBE Score	Type of sample	Nationality
Amirmozafari [17]	Tehran	Spoligo	2006	CAS- EAI-Beijing-T- Haarlem-MANU	CAS:19- EAI:31-Beijing:14-T:39- Haarlem:75-MANU:3-LAM:1-West African:1	–	439	23	PTB-EPTB	Iranian - Afghan immigrants
Ramazanzadeh [18]	Tehran	Spoligo	2006	CAS- EAI-Beijing -T- Haarlem-WestAfrican - LAM	CAS:7- EAI:27-Beijing:12-T:36- Haarlem:65-WestAfrican:1- LAM:1	–	346	26	PTB-EPTB	Iranian
Ramazanzadeh [18]	Tehran	Spoligo	2006	CAS- EAI-Beijing -T- Haarlem	CAS:1- EAI:4-Beijing: 5-T:6- Haarlem:20	–	93	27	PTB-EPTB	Afghan immigrants
Masjedi [19]	Tehran	IS6110-RFLP /Spoligo	2008	CAS- EAI-Beijing-T- Haarlem-X	CAS:38-EAI:71-Beijing:8-T:44-Haarlem:14-X:1	Beijing:2-Haarlem:3	199	26	PTB-EPTB	Iranian
Masjedi [19]	Tehran	IS6110-RFLP /Spoligo	2008	CAS- EAI-Beijing-T- Haarlem-X	CAS:12-EAI:19-Beijing:4-T:3-Haarlem:6-X:11	Beijing:1-T:1-Haarlem:5	59	24	PTB-EPTB	Afghan immigrants
Tajeddin [20]	Tehran	Spoligo	2009	CAS- EAI-Beijing-T- Haarlem-U	CAS:118-EAI:64-Beijing:15-T:16-Haarlem:13-U:12	CAS:7-EAI:8-Beijing:6-T:2- U:2-HaarlemI:3	238	28	PTB	Iranian
Ramazanzadeh [21]	Tehran	Spoligo	2009	CAS-Beijing-T-Haarlem- MANU-EAI-LAM-West African	CAS:18-Beijing:14-T:39-Haarlem:75-MANU:4- EAI:31-LAM:1-West African:1	–	523	30	PTB-EPTB	Iranian - Afghan immigrants
Rohani [22]	Mashhad	Spoligo	2009	CAS-NEW1-Beijing-T-Haarlem- MANU-URAL-EASTMED	CAS:6-NEW1:2-Beijing:8-T:2-Haarlem:1-MANU:14-URAL:1-EASTMED:3	Beijing:3	113	25	PTB	Iranian - Afghan immigrants
Ahmadi [23]	Tehran	Spoligo	2009	CAS- EAI-Beijing-T- Haarlem-U-Bovis	CAS:55- EAI:39-Beijing:6-T:16- Haarlem:52-U:9-Bovis:2	–	179	24	PTB	Iranian
Ahmadi [23]	Tehran	Spoligo	2009	CAS- EAI-Beijing- Haarlem-U	CAS:19- EAI:16-Beijing:7- Haarlem:14-U:3	–	59	22	PTB	Afghan immigrants
Jafarian [24]	Tehran	12 loci MIRU-VNTR /Spoligo	2010	CAS- NEW1-Haarlem-LAM-Uganda	CAS:8- NEW1:6-Haarlem:7-LAM:2-Uganda:2	0	30	24	PTB	Iranian
Merza [25]	Tehran	Spoligo	2010	CAS- Beijing- EAI- Haarlem-T- X	CAS:169- Beijing: 11- EAI:212- Haarlem:41, T:324-X:16	–	981	30	PTB	Iranian
Merza [25]	Tehran	Spoligo	2010	CAS- Beijing- EAI- Haarlem-T- X	CAS:176- Beijing: 76- EAI:174- Haarlem:31, T:67-X:10	–	634	30	PTB	Afghan immigrants
Merza [25]	Tehran	Spoligo	2010	CAS- Beijing- EAI- Haarlem-T- X	CAS:12- Beijing: 1- EAI:15- Haarlem:3- T:23-X:2	–	93	22	EPTB	Iranian
Merza [25]	Tehran	Spoligo	2010	CAS- Beijing- EAI- Haarlem-T- X	CAS:7- Beijing: 4- EAI:5- Haarlem:2- T:3-X:1	–	34	23	EPTB	Afghan immigrants
Asgharzadeh [26]	Azarbaijan	IS6110-RFLP /12 loci MIRU-VNTR	2011	NEW1-Haarlem-S-Uganda-X- Cameroon-Bovis	NEW1:1-Haarlem:1-S:13-Uganda:1-X:1-Cameroon:1-Bovis:15	–	154	26	PTB-EPTB	Iranian
Jafarian [27]	Tehran	12 loci MIRU-VNTR	2011	CAS- NEW1-Beijing-Haarlem-EAI-LAM-Uganda-H37Rv-Bovis	CAS:49- NEW1:16- Beijing:14-Haarlem:3-EAI:1-LAM:14-Uganda:28-H37Rv:5-Bovis:10	–	140	26	PTB	Iranian - Afghan immigrants
Zaker Bostanabad [28]	Tehran	Spoligo	2011	CAS- Beijing- EAI- Haarlem-T- U	CAS:18- Beijing: 38- U:8-EAI:34- Haarlem:41- T:10	CAS:2-Beijing:1-EAI:3-U:1	149	25	PTB	Iranian
Mozafari [29]	Tehran	12/15 loci MIRU-VNTR /Spoligo	2012	CAS-NEW1-Beijing-Haarlem-LAM-URAL-Uganda-X-S-Camroon-Bovis	CAS:22-NEW1:25-Beijing:20-Haarlem:2- LAM:5-URAL:3-Uganda:9-X:1-S:2-Cameroon:1-Bovis:1	Beijing:7	105	27	PTB	Iranian
Haeili [30]	5 provinces	Spoligo	2013	CAS- NEW1- Beijing- Haarlem-T-MANU-LAM-EAI-U-H37Rv	CAS:70-NEW1:100-Beijing: 3-T:53-Haarlem:3- MANU:22-LAM:18-EAI:4-U:3-H37Rv:2	CAS:1- NEW1:5-Beijing: 3	291	29	PTB-EPTB	Iranian
Torkaman [31]	Tehran	15 loci MIRU-VNTR /Spoligo	2014	CAS-NEW1-Beijing-Haarlem-URAL-T-MANU-Bovis	CAS:15-NEW1:14-Beijing:6-Haarlem:3- T:6-MANU:8-URAL:2-Bovis:3	–	73	26	PTB	Iranian
Torkaman [31]	Tehran	15 loci MIRU-VNTR /Spoligo	2014	CAS-NEW1-Beijing-Haarlem	CAS:7-NEW1:7-Beijing:5-Haarlem:1	–	29	24	PTB	Afghan immigrants
Velayati [32]	21 provinces	Spoligo	2014	CAS-Beijing- Haarlem-T-MANU-LAM-EAI-U-X- Bovis	CAS:471-EAI:3-Beijing: 101-T:195-Haarlem:326- MANU:25-LAM:15-U:17-Bovis:10-X:2	–	1242	31	PTB	Iranian- immigrants
Varahram [33]	Tehran	Spoligo	2014	CAS-Beijing- Haarlem-T-MANU-LAM-EAI-U	CAS:23-EAI:42-Beijing: 14-T:11-Haarlem:31- MANU:9-LAM:3-U:7	CAS:1- T:1-EAI:5-Beijing:10- Haarlem:2- MANU:2-LAM:1-U:1	151	27	PTB	Iranian - Afghan immigrants
Sharifpour [34]	Tehran	Spoligo	2014	CAS-Beijing- Haarlem-T	CAS:37-Beijing:11-Haarlem:72-T:18	CAS:3-T:3-Beijing:4- Haarlem:3	190	25	PTB	Iranian
Haeili [35]	IRAN	Spoligo	2015	CAS-NEW1-T-MANU-LAM	CAS:60-NEW1:86- T:46- MANU:19-LAM:15	–	251	25	PTB	Iranian
Sharifpour [36]	Tehran	RD Typing/Spoligo	2016	CAS-Beijing- Haarlem-T	CAS:54-Beijing:28- Haarlem:85-T:27	–	250	26	PTB	Iranian
Feyisa [37]	Tehran	IS6110-RFLP /Spoligo	2016	CAS- NEW1- Beijing-Haarlem-T-EAI-MANU-LAM-H37Rv	CAS:18- NEW1:15- Beijing:1-Haarlem:1-T:7- EAI:4-MANU:3-LAM:1- H37Rv:1	–	60	22	PTB	Iranian
Feyisa [37]	Tehran	IS6110-RFLP /Spoligo	2016	NEW1-T-EAI	NEW1:7-T:2- EAI:1	–	10	20	PTB	Afghan immigrants
Zamani [38]	Hormozgan	15loci MIRU-VNTR/Spoligo	2016	CAS-Beijing-NEW1-T-Haarlem-LAM-MANU-EAI	CAS:7-Beijing:1-NEW1:2- Haarlem:1-LAM:9- MANU:3-EAI:1-T:5	T:2-MANU:1	38	25	PTB	Iranian
Riyahi Zaniani [39]	Isfahan	15loci MIRU-VNTR	2017	CAS- NEW1- Beijing-URAL- LAM-S-X-EAI	CAS:10- NEW1:9- Beijing: 9-URAL:2- LAM:3-S:3-X: 1-EAI:1	–	38	24	PTB	Iranian
Riyahi Zaniani [39]	Isfahan	15loci MIRU-VNTR	2017	CAS- NEW1- Beijing-URAL- LAM-S-X-EAI	CAS:4- NEW1:3- Beijing: 3- S:1	–	11	23	PTB	Afghan immigrants
Ravansalar [40]	Khorasan	12 loci MIRU-VNTR /Spoligo	2017	CAS- NEW1- Beijing-T-Haarlem-MANU-U	CAS:19-Beijing:9-T:3-Haarlem:67-MANU:1-U:5	Beijing:2	140	25	PTB	Iranian
Mansoori [41]	Golestan	24 loci MIRU-VNTR	2018	CAS- NEW1- Beijing-URAL- Haarlem-TUR	CAS:31-NEW1:45-Beijing:18-URAL:4-TUR:2-Haarlem:2	0	156	25	PTB-EPTB	Iranian
Azimi [42]	Tehran	15loci MIRU-VNTR	2018	CAS- NEW1 -WestAfrican - Bovis- H37Rv	CAS:1 -NEW1:18-WestAfrican:1 - Bovis: 1- H37Rv: 1	–	80	24	PTB	Iranian
Kargarpour [43]	Tehran	Spoligo	2018	CAS-NEW1-T-MANU	CAS:2-NEW1:3-T:1-MANU:5	–	14	25	PTB-EPTB	Iranian
Kochkaksaraei [44]	Golestan	15loci MIRU-VNTR	2019	CAS- NEW1- Beijing-URAL-TUR-Cameroon	CAS:15-NEW1:36- Beijing:22-URAL:3-TUR:1-Cameroon:4	0	162	28	PTB-EPTB	Iranian
Hadifar [45]	Tehran	24 loci MIRU-VNTR /Spoligo	2019	CAS- NEW1- Beijing-URAL- T-Haarlem-EASTMED	CAS:25-NEW1:21-Beijing:10-URAL:4-T:17-Haarlem:2-EASTMED:4	CAS:1-NEW1:1-Beijing:5-T:1	84	27	PTB	Iranian
Hadifar [45]	Tehran	24 loci MIRU-VNTR /Spoligo	2019	CAS- NEW1- Beijing-URAL- T-Haarlem-EASTMED	CAS:21-NEW1:28-Beijing:5-URAL:6-T:22-Haarlem:2-EASTMED:1-U:2	CAS:1-NEW1:1	88	28	EPTB	Iranian
Afaghi-Gharamaleki [46]	Tabriz	15loci MIRU-VNTR	2019	CAS- NEW1 -Beijing-Uganda-LAM-TUR-Cameroon- Bovis- H37Rv	CAS:1- NEW1:30- Beijing:4-Uganda:18-LAM:1-TUR:2-Cameroon:1- Bovis:3- H37Rv:1	–	91	27	PTB	Iranian
Hadifar [47]	Tehran	24 loci MIRU-VNTR /Spoligo	2019	CAS- NEW1- Beijing	CAS:95-NEW1:80-Beijing:42	–	217	29	PTB	Iranian
Vaziri [48]	Tehran	WGS	2019	CAS- NEW1- Beijing-URAL- T-TUR-H37Rv	CAS:7-NEW1:8-Beijing:14- URAL:2- T:4- TUR:1- H37Rv:1	Beijing:14	38	25	PTB	Iranian
Kargarpour [49]	Tehran	24 loci MIRU-VNTR	2019	CAS- NEW1- Beijing- Haarlem-Uganda	CAS:5-NEW1:2-Beijing:2-Haarlem:1-Uganda:1	–	12	26	PTB	Iranian
Kargarpour [50]	Tehran	24 loci MIRU-VNTR	2020	CAS- NEW1- Beijing- Haarlem-Uganda	CAS:9-NEW1:4-Beijing:5-Haarlem:1-Uganda:1	0	45	28	PTB	Iranian

*PTB* Pulmonary Tuberculosis, *EPTB* Extrapulmonary Tuberculosis, *WGS* Whole-Genome Sequencing, *MDR* Multidrug resistant

### Statistical analysis

Pooled proportion and 95% CI were used to assess the prevalence of the genotypes in the pulmonary tuberculosis (PTB) and extrapulmonary tuberculosis (EPTB) samples. Generalized linear mixed model with random intercept logistic regression model was used for assessing pooled prevalence [52]. The heterogeneity of prevalence between the included studies was tested and quantified by using Cochran’s Q test and I^2^ index, respectively [53]. Clopper-Pearson was run for evaluating pooled proportion and confidence interval in the individual studies. Also, continuity correction of 0.5 was considered in studies with zero cell frequencies [54]. The pooled proportion, as an overall prevalence of the genotypes, was derived by the random effects model because of significant heterogeneity between the individual studies. Publication bias was tested by Egger’s linear regression test and Begg’s test (*P* < 0.05 was set as the significance level for publication bias) [55]. All the statistical analyses were performed by using the metafor R package and MedCalc software.

## Results

### Search results and studies’ characteristics

**Fig. 1:**
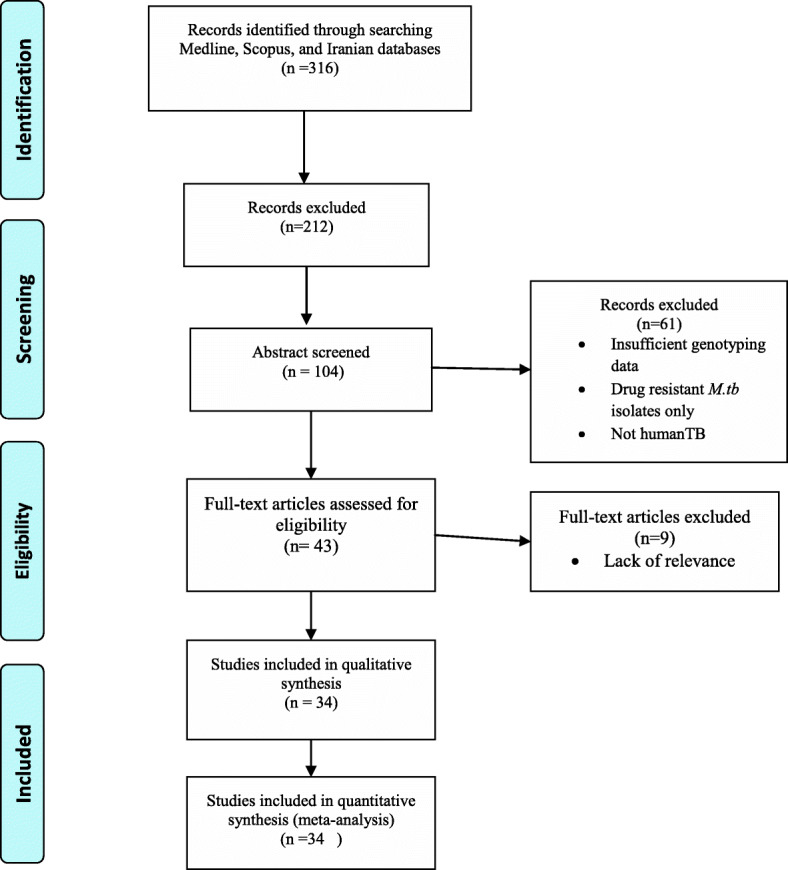
Flow diagram of literature selection process in the meta-analysis

A total of 316 articles were identified by the primary search strategy, of which 34 articles met the eligibility criteria and were included in this study (Fig. 1). The selected studies included 8329 clinical samples. Most of the studies were conducted in Tehran (capital of Iran). Publication year of these studies ranged from 2006 to 2020. Spoligotyping and MIRU-VNTR typing were identified as the most common methods of genotyping.

### Quality assessment

Based on the scores of the STROBE checklist, the highest and lowest scores were related to the studies of Velayati et al. (2014) and Feyisa et al. (2016), respectively. The mean score of STROBE tool was 25.72 (SD = 2.42, range: 20–31) (Table 1).

### Pooled prevalence of MTBC genotypes in the PTB and EPTB samples

**Fig. 2:**
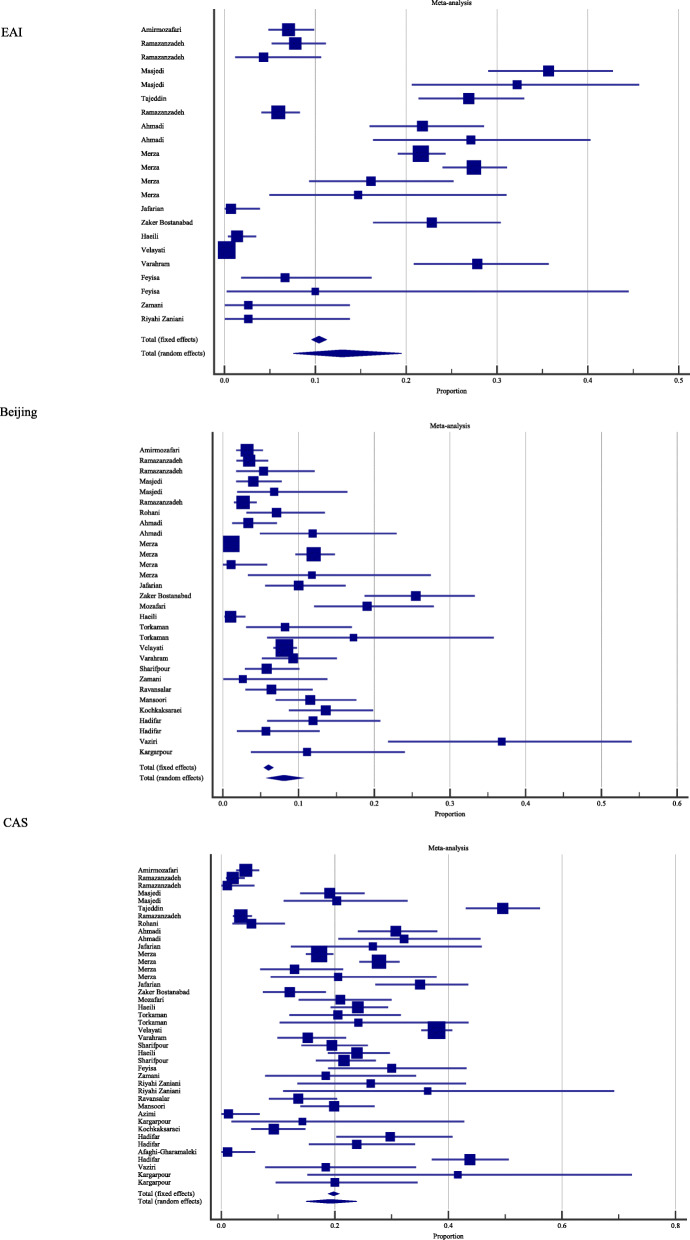
Forest plots displaying the prevalence of different *M.tb* genotypes in the studied geographical region

Results of the random or fixed effects meta-analysis are summarized in Table 2. *M. bovis* as a member of the animal-adapted MTBC accounted for only 3.29% of the studied strains and *Mtb* sensu stricto (Lineages1–4) comprised the largest proportion of the studied strains. Based on the pooled prevalence of the *Mtb* genotypes in the PTB and EPTB samples, NEW1 (21.94, 95% CI: 16.41–28.05%), CAS (19.21, 95% CI: 14.95–23.86%), EAI (12.95, 95% CI: 7.58–19.47%), and T (12.16, 95% CI: 9.18–15.50%) were found to be the dominant circulating genotypes in Iran. West African (L 5/6), Cameroon, TUR and H37Rv (parts of the Euro-American super-lineage [L4]) were identified as genotypes with the lowest prevalence in Iran (< 2%). The forest plot of some of the genotypes (i.e., Beijing, CAS, and EAI) are shown in Fig. 2. In addition, the highest pooled prevalence of MDR strains was found in Beijing (2.52,95% CI) and CAS (1.21,95% CI) genotypes (Table 2).

**Table 2:** Pooled prevalence of MTBC genotypes in each studied genotype in PTB and EPTB samples

Lineage	Genotype	(n), Pooled prevalence of MDR%	Pooled prevalence of genotype		Heterogeneity		Publication bias	
			(n), Prevalence%	(95% CI)%	Q, I^2^%	*P*-value	Begg’s *p-*value	Egger’s *p-*value
L1	**EAI**	(16), 1.17%	(799), **12.95%**	(7.58–19.47)%	935, 97.75%	< 0.001	0.061	0.192
	**MANU**	(3), 0.74%	(116), 5.41%	(3.03–8.43)%	105, 89.58%	< 0.001	0.06	0.17
L2	**Beijing**	(52), **2.52%**	(481), 8.06%	(5.96–10.44)%	305, 90.52%	< 0.001	0.14	0.27
L3	**CAS**	(23), **1.21%**	(1761), **19.21%**	(14.95–23.86)%	1016, 95.97%	< 0.001	0.11	0.16
L4	**NEW1**	(7), 0.8%	(568), **21.94%**	(16.41–28.05)%	263, 90.89	< 0.001	0.09	0.31
	**T**	(10), 0.59%	(1006), **12.16%**	(9.18–15.50)%	367, 92.65%	< 0.001	0.089	0.216
	**Haarlem**	(13), 0.67%	(989), 10.38%	(6.62–14.87)%	909, 96.59%	< 0.001	0.054	0.116
	**Uganda**	0%	(60), 9.04%	(3.06–17.74)%	53, 88.87%	< 0.001	0.24	0.64
	**S**	0%	(19) 6.24%	(3.83–9.53)%	6, 53.57%	0.091	0.415	0.817
	**LAM**	(1), 0.31%	(89), 3.59%	(1.85–5.86)%	119, 89.08%	< 0.001	0.052	0.095
	**URAL**	0%	(27), 3.39%	(2.28–4.82)%	8.49, 5.83%	0.386	0.612	0.768
	**EASTMED**	0%	(8), 3.12%	(1.43–5.84)%	2, 0%	0.393	0.78	0.81
	**X**	0%	(46), 2.03%	(0.88–3.64)%	55, 83.76%	< 0.001	0.153	0.319
	**H37Rv**	0%	(11), 1.78%	(0.94–3.05)%	5, 1.99%	0.403	0.419	0.778
	**TUR**	0%	(6), 1.64%	(0.68–3.29)%	1.91, 0%	0.59	0.419	0.654
	**Cameroon**	0%	(7), 1.64%	(0.73–3.15)%	1.61, 0%	0.656	0.69	0.78
L5/6	**West African**	0%	(4), 0.38%	(0.13–0.87)%	1.9, 0%	0.586	0.513	0.813
	**Bovis**	0%	(45), 3.29%	(1.31–6.13)%	45, 84.73%	< 0.001	0.112	0.278
	**U**	(4), 0.53%	(66), 3.55%	(2.13–5.31)%	28, 72.14%	< 0.001	0.09	0.26

### Publication bias

We observed significant heterogeneity across the studies based on the I^2^ index with a few exceptions (Table 2). However, publication bias was not significant based on the results of Egger’s linear regression test and Begg’s test.

## Discussion

Based on the pooled data investigated, all MTBC lineages, except lineage 7 and 8, were found in Iran, which reflects the presence of high diversity in MTBC strains. Phylogeographical population structure of the MTBC stems from the interplay between different factors such as human migration, geography, genetic drift and host-pathogen interaction [4, 5, 56]. Iran is the main host country for Afghan refugees, but the main factor contributing to formation of MTBC lineages phylogeography in Iran has not been identified.

Study of global variation in MTBC strains showed that the prevalence of lineages 2, 3 and 4 strains may be increasing in West Asia, while the prevalence of lineage 1 is declining [15]. The summary of *Mtb* strains diversity in Iran, based on families/sublineage, showed that NEW1(L4) (21.94, 95% CI: 16.41–28.05%), CAS (L3) (19.21, 95% CI: 14.95–23.86%), EAI(L1) (12.95, 95% CI: 7.58–19.47%), and T (L4) (12.16, 95% CI: 9.18–15.50%) were the dominant circulating *Mtb* genotypes. EAI (L1) and CAS (L3) are mainly confined to the areas around the Indian Ocean [11]. Movement of strains with people from these regions may explain the presence of these genotypes in Iran. Besides, appearing CAS as a one of the prevalent *Mtb* subpopulations in Iran may reflect the pathogenic properties of this genotype.

In a recent study, the global proportion of MDR in CAS population was estimated at 30.63% [57]. In our study, based on the pooled prevalence of MDR genotype, CAS was found (1.21%) as a one of the dominant genotypes. This finding reflects the needs for more understanding and monitoring of this subpopulation.

Despite the global dissemination of Beijing genotype as a prototype of lineage 2, it had low prevalence in our geographical region. However, the highest pooled prevalence of MDR strains was found in the Beijing (2.52%) genotype. This result is consistent with the previously published reports about the prevalence of Beijing among MDR-TB isolates in Iran [58]. The low prevalence of Beijing genotype compared to other genotypes in Iran may be explained by the prevalent Beijing sublineage, affecting its pathobiological properties and epidemiological dynamics. Further studies are warranted to identify the distribution pattern of the Beijing sublineages in Iran, which can improve the management of their infection.

The dominance of NEW1 as a specialist sublineage of Euro-American lineage (L4) in Iran was not unexpected. Some evidence has shown that Iran is the probable origin of this family/sublineage, which may reflect ecological adaption in this subpopulation [59]. It is noteworthy that NEW1 genotype is prone to MDR [13]. The pooled prevalence of MDR in NEW1 was 0.8% (95% CI). However, the results of overall MDR estimation may be less representative of the target population, as in the some of the included studies in our analysis; drug susceptibility testing was not reported based on the identified genotype, which may lead to variation in the final results. Other sublineages of lineage 4 such as T, Haarlem, Uganda and S in varying proportions were also observed. This distribution pattern in the subtypes of lineage 4 in Iran may be explained by the effect of human migration and genetic and phenotypic characteristics of each sublineage.

In addition, we observed that lineage 5/6 subtype had the lowest prevalence in our geographical region. Based on the fact that these strains are geographically restricted [2], we can only speculate human migration as the determinant of this distribution. A limitation of this study is that most of the included studies were conducted in Tehran (Capital of Iran). Thus, our finding may not be completely representative of the overall prevalence of different *Mtb* populations in Iran. In addition, the most of the included studies were based on Spoligotyping and MIRU-VNTR typing methods while WGS provides a superior resolution compared with these PCR-based genotyping methods to identification of diversity in *Mtb* strains.

## Conclusions

In summary, this systematic review showed that *Mtb* population are genetically diverse in Iran and the NEW1 (L4) and West African (L5/6) genotypes had the highest and lowest pooled prevalence rates, respectively. This type of evidence can contribute to better clinical and epidemiological management of *Mtb* infections. Also, there is a need for further in-depth studies to gain a deeper insight into the national diversity of *Mtb* populations and their drug resistance pattern.

## Data Availability

All data generated or analysed during this study are included in this published article and its supplementary information files.

## Abbreviations

MTBC: *Mycobacterium tuberculosis* complex
*Mtb*: *Mycobacterium tuberculosis*
MIRU-VNTR: mycobacterial interspersed repetitive unit-variable number tandem repeat
TB: Tuberculosis
MDR: multidrug-resistant
PRISMA: Preferred Reporting Items for Systematic Reviews and Meta-Analyses
STROBE: Strengthening the Reporting of Observational Studies in Epidemiology
PTB: Pulmonary tuberculosis
EPTB: Extrapulmonary tuberculosis
WGS: Whole-Genome Sequencing

## References

[1] WHO (2019). WHO global report, global tuberculosis report 2019.

[2] Brites D, Gagneux S (2017). The nature and evolution of genomic diversity in the mycobacterium tuberculosis complex. Adv Exp Med Biol.

[3] Ngabonziza JCS, Loiseau C, Marceau M, Jouet A, Menardo F, Tzfadia O (2020). A sister lineage of the mycobacterium tuberculosis complex discovered in the African Great Lakes region. Nat Commun.

[4] Comas I, Coscolla M, Luo T, Borrell S, Holt KE, Kato-Maeda M (2013). Out-of-Africa migration and Neolithic coexpansion of mycobacterium tuberculosis with modern humans. Nat Genet.

[5] Gagneux S, DeRiemer K, Van T, Kato-Maeda M, De Jong BC, Narayanan S (2006). Variable host–pathogen compatibility in mycobacterium tuberculosis. Proc Natl Acad Sci.

[6] Hadifar S, Behrouzi A, Fateh A, Khatami S, Rahimi Jamnani F, Siadat SD (2019). Comparative study of interruption of signaling pathways in lung epithelial cell by two different mycobacterium tuberculosis lineages. J Cell Physiol.

[7] Chapman HJ, Phillips SA, Hosford JL, Séraphin MN, Lauzardo M (2015). Is the Beijing strain of mycobacterium tuberculosis associated with cavitary lung disease?. Infect Genet Evol.

[8] Sarkar R, Lenders L, Wilkinson KA, Wilkinson RJ, Nicol MP (2012). Modern lineages of mycobacterium tuberculosis exhibit lineage-specific patterns of growth and cytokine induction in human monocyte-derived macrophages. PLOS ONE..

[9] Hanekom M, Van Pittius NG, McEvoy C, Victor T, Van Helden P, Warren R (2011). Mycobacterium tuberculosis Beijing genotype: a template for success. Tuberculosis.

[10] Brynildsrud OB, Pepperell CS, Suffys P, Grandjean L, Monteserin J, Debech N (2018). Global expansion of mycobacterium tuberculosis lineage 4 shaped by colonial migration and local adaptation. Sci Adv.

[11] Coscolla M, Gagneux S (2017). Biological and epidemiological consequences of MTBC diversity. Strain variation in the Mycobacterium tuberculosis complex: its role in biology, epidemiology and control.

[12] Stucki D, Brites D, Jeljeli L, Coscolla M, Liu Q, Trauner A (2016). Mycobacterium tuberculosis lineage 4 comprises globally distributed and geographically restricted sublineages. Nat Genet.

[13] Mokrousov I (2016). Emerging resistant clone of mycobacterium tuberculosis in West Asia. Lancet Infect Dis.

[14] Yasmin M, Gomgnimbou MK, Siddiqui RT, Refrégier G, Sola C (2014). Multi-drug resistant mycobacterium tuberculosis complex genetic diversity and clues on recent transmission in Punjab, Pakistan. Infect Genet Evol.

[15] Wiens KE, Woyczynski LP, Ledesma JR, Ross JM, Zenteno-Cuevas R, Goodridge A (2018). Global variation in bacterial strains that cause tuberculosis disease: a systematic review and meta-analysis. BMC Med.

[16] Moher D, Liberati A, Tetzlaff J, Altman DG (2009). Preferred reporting items for systematic reviews and meta-analyses: the PRISMA statement. Ann Intern Med.

[17] Amirmozafari N, Ramezanzadeh R, Farnia P, Ghazi F (2006). The frequency of Beijing genotype of mycobacterium tuberculosis isolated from tuberculosis patients. Razi J Med Sci.

[18] Ramazanzadeh R, Amirmozafari N, Farnia P, Ghazi F (2006). Genotyping of mycobacterium tuberculosis isolates from TB patients with spoligotyping. Scie J Kurdistan Univ Med Sci.

[19] Masjedi MR, Varahram M, Mirsaeidi M, Ahmadi M, Khazampour M, Tabarsi P (2008). The recent-transmission of mycobacterium tuberculosis strains among Iranian and afghan relapse cases: a DNA-fingerprinting using RFLP and spoligotyping. BMC Infect Dis.

[20] Tajeddin E, Farnia P, Kargar M, Noroozi J, Ahmadi M, Kazempour M (2009). Identification of mycobacterium tuberculosis Beijing genotype using three different molecular methods. Koomesh..

[21] Ramazanzadeh R, Farnia P, Amirmozafari N (2009). Characterization of mycobacterium tuberculosis complex isolated from iranian and afghani patients by spoligotyping method. Braz J Microbiol.

[22] Rohani M, Farnia P, Nasab MN, Moniri R, Torfeh M, Amiri M (2009). Beijing genotype and other predominant mycobacterium tuberculosis spoligotypes observed in Mashhad city, Iran. Indian J Med Microbiol.

[23] Ahmadi M, Farnia P, Tajedin E, Tabarsi P, Baghaei P, Masjedi M (2009). Mycobacterium tuberculosis complex strains identification with Spoligotyping method in patients attending to Masih Daneshvari hospital. J Adv Med Biomed Res.

[24] Jafarian M, Aghali-Merza M, Farnia P, Ahmadi M, Masjedi MR, Velayati AA (2010). Synchronous comparison of mycobacterium tuberculosis epidemiology strains by “MIRU-VNTR” and “MIRU-VNTR and Spoligotyping” technique. Avicenna J Med Biotechnol.

[25] Merza MA, Farnia P, Salih AM, Masjedi MR, Velayati AA (2010). The most predominant spoligopatterns of mycobacterium tuberculosis isolates among Iranian, afghan-immigrant, Pakistani and Turkish tuberculosis patients: a comparative analysis. Chemotherapy.

[26] Asgharzadeh M, Kafil HS, Roudsary AA, Hanifi GR (2011). Tuberculosis transmission in northwest of Iran: using MIRU-VNTR, ETR-VNTR and IS6110-RFLP methods. Infect Genet Evol.

[27] Jafarian M, Farnia P, Mozafari M, Ahmadi M, Masjiedi M, Velayati A (2011). Comparison of the genetic convergence degree of mycobacterium tuberculosis (TB) strains isolated from patients infected with TB by MIRU-VNTR technique. J Adv Med Biomed Res.

[28] Zaker Bostanabad S, Jabbarzadeh E, Pourazar S, Ghalami M (2011). Typing of mycobacterium tuberculosis isolated from patients in the Tehran City by Spoilgotyping. New Cellular Mol Biotechnol J.

[29] Mozafari M, Farnia P, Jafarian M, Razavi Deligani M, Kazempour M, Masjedi M (2012). Comparison of mycobacterium tuberculosis Beijing genotype with other mycobacterium tuberculosis strains using MIRU-VNTR method. ISMJ.

[30] Haeili M, Darban-Sarokhalil D, Fooladi AAI, Javadpour S, Hashemi A, Siavoshi F (2013). Spoligotyping and drug resistance patterns of mycobacterium tuberculosis isolates from five provinces of Iran. Microbiologyopen.

[31] Torkaman MRA, Nasiri MJ, FaRnia P, Shahhosseiny MH, Mozafari M, Velayati AA (2014). Estimation of recent transmission of mycobacterium tuberculosis strains among Iranian and afghan immigrants: a cluster-based study. J Clin Diagn Res.

[32] Velayati AA, Farnia P, Mozafari M, Sheikholeslami MF, Karahrudi MA, Tabarsi P (2014). High prevelance of rifampin-monoresistant tuberculosis: a retrospective analysis among Iranian pulmonary tuberculosis patients. Am J Trop Med Hyg.

[33] Varahram M, Farnia P, Nasiri MJ, Karahrudi MA, Dizagie MK, Velayati AA. Association of Mycobacterium tuberculosis lineages with IFN-γ and TNF-α gene polymorphisms among pulmonary tuberculosis patient. Mediterranean J Hematol Infect Dis. 2014;6(1):e2014015–e. 10.4084/MJHID.2014.015 PMC3965727 24678392

[34] Sharifipour E, Nasiri M, Farnia P, Mozafari M, Irani S (2014). Evaluation of molecular diversity of mycobacterium tuberculosis strains by polymorphisms in RD regions. J Mycobac Dis.

[35] Haeili M, Darban-Sarokhalil D, Fooladi AI, Zamani S, Zahednamazi F, Kardan J (2015). Genotyping and drug susceptibility testing of mycobacterium tuberculosis isolates from Iran. Int J Mycobacteriol.

[36] Sharifipour E, Farnia P, Mozafari M, Irani S, Velayati AA (2016). Deletion of region of difference 181 in mycobacterium tuberculosis Beijing strains. Int J Mycobacteriol.

[37] Feyisa SG, Haeili M, Zahednamazi F, Mosavari N, Taheri MM, Hamzehloo G (2016). Molecular characterization of mycobacterium tuberculosis isolates from Tehran, Iran by restriction fragment length polymorphism analysis and spoligotyping. Rev Soc Bras Med Trop.

[38] Zamani S, Haeili M, Nasiri MJ, Imani Fooladi AA, Javadpour S, Feizabadi MM (2016). Genotyping of mycobacterium tuberculosis isolates from Hormozgan Province of Iran based on 15-locus MIRU-VNTR and Spoligotyping. Int J Bacteriol.

[39] Zaniani FR, Moghim S, Mirhendi H, Safaei HG, Fazeli H, Salehi M (2017). Genetic lineages of mycobacterium tuberculosis isolates in Isfahan, Iran. Curr Microbiol.

[40] Ravansalar H, Tadayon K, Mosavari N, Derakhshan M, Ghazvini K (2017). Genetic diversity of mycobacterium tuberculosis complex isolated from patients in the northeast of Iran by MIRU-VNTR and spoligotyping. Jundishapur J Microbiol..

[41] Mansoori N, Yaseri M, Vaziri F, Douraghi M (2018). Genetic diversity of mycobacterium tuberculosis complex isolates circulating in an area with high tuberculosis incidence: using 24-locus MIRU-VNTR method. Tuberculosis.

[42] Azimi T, Nasiri MJ, Zamani S, Hashemi A, Goudarzi H, Fooladi AAI (2018). High genetic diversity among mycobacterium tuberculosis strains in Tehran, Iran. J Clin Tubercul Mycobacterial Dis.

[43] Kamakoli MK, Khanipour S, Hadifar S, Ghajavand H, Farmanfarmaei G, Fateh A (2018). Challenge in direct Spoligotyping of mycobacterium tuberculosis: a problematic issue in the region with high prevalence of polyclonal infections. BMC Res Notes.

[44] Babai Kochkaksaraei M, Kaboosi H, Ghaemi EA (2019). Genetic variation of the mycobacterium tuberculosis in north of Iran; the Golestan Province. Iran Red Crescent Med J..

[45] Hadifar S, Shamkhali L, Kamakoli MK, Mostafaei S, Khanipour S, Mansoori N (2019). Genetic diversity of mycobacterium tuberculosis isolates causing pulmonary and extrapulmonary tuberculosis in the capital of Iran. Mol Phylogenet Evol.

[46] Afaghi GA, Moaddab SR, Darbouy M, Ansarian K, Hanifian S (2019). Genotypic diversity of resistant mycobacterium tuberculosis strains isolated from tuberculosis patients in East Azerbaijan center by MIRU-VNTR. J Microb World.

[47] Hadifar S, Kamakoli MK, Fateh A, Siadat SD, Vaziri F (2019). Enhancing the differentiation of specific genotypes in mycobacterium tuberculosis population. Sci Rep.

[48] Vaziri F, Kohl TA, Ghajavand H, Kargarpour Kamakoli M, Merker M, Hadifar S (2019). Genetic diversity of multi- and extensively drug-resistant mycobacterium tuberculosis isolates in the Capital of Iran, revealed by whole-genome sequencing. J Clin Microbiol.

[49] Kargarpour Kamakoli M, Hadifar S, Khanipour S, Farmanfarmaei G, Fateh A, Siadat SD (2019). Comparison of MIRU-VNTR genotyping between old and fresh clinical samples in tuberculosis. Infect Dis.

[50] Kamakoli MK, Farmanfarmaei G, Masoumi M, Khanipour S, Gharibzadeh S, Sola C (2020). Prediction of the hidden genotype of mixed infection strains in Iranian tuberculosis patients. Int J Infect Dis.

[51] Von Elm E, Altman DG, Egger M, Pocock SJ, Gøtzsche PC, Vandenbroucke JP (2007). The strengthening the reporting of observational studies in epidemiology (STROBE) statement: guidelines for reporting observational studies. Ann Intern Med.

[52] Stijnen T, Hamza TH, Özdemir P (2010). Random effects meta-analysis of event outcome in the framework of the generalized linear mixed model with applications in sparse data. Stat Med.

[53] Huedo-Medina TB, Sánchez-Meca J, Marín-Martínez F, Botella J (2006). Assessing heterogeneity in meta-analysis: Q statistic or I^2^ index?. Psychol Methods.

[54] Viechtbauer W (2010). Conducting meta-analyses in R with the metafor package. J Stat Softw.

[55] Egger M, Smith GD, Schneider M, Minder C (1997). Bias in meta-analysis detected by a simple, graphical test. Bmj.

[56] Hershberg R, Lipatov M, Small PM, Sheffer H, Niemann S, Homolka S (2008). High functional diversity in mycobacterium tuberculosis driven by genetic drift and human demography. PLoS Biol..

[57] Couvin D, Reynaud Y, Rastogi N (2019). Two tales: worldwide distribution of central Asian (CAS) versus ancestral east-African Indian (EAI) lineages of mycobacterium tuberculosis underlines a remarkable cleavage for phylogeographical, epidemiological and demographical characteristics. PLoS One..

[58] Tarashi S, Fateh A, Jamnani FR, Siadat SD, Vaziri F (2017). Prevalence of Beijing and Haarlem genotypes among multidrug-resistant mycobacterium tuberculosis in Iran: systematic review and meta-analysis. Tuberculosis.

[59] Mokrousov I, Shitikov E, Skiba Y, Kolchenko S, Chernyaeva E, Vyazovaya A (2017). Emerging peak on the phylogeographic landscape of mycobacterium tuberculosis in West Asia: definitely smoke, likely fire. Mol Phylogenet Evol.

